# Altered resting state functional connectivity in youth with congenital heart disease operated during infancy

**DOI:** 10.1371/journal.pone.0264781

**Published:** 2022-04-15

**Authors:** Vincente Enguix, Kaitlyn Easson, Guillaume Gilbert, Christine Saint-Martin, Charles Rohlicek, David Luck, Gregory Anton Lodygensky, Marie Brossard-Racine

**Affiliations:** 1 Canadian Neonatal Brain Platform, Montreal, Canada; 2 Department of Pediatrics, CHU Sainte-Justine Research Center, University of Montreal, Montreal, Canada; 3 Advances in Brain & Child Development (ABCD) Research Laboratory, Research Institute of the McGill University Health Centre, Montreal, QC, Canada; 4 Department of Neurology & Neurosurgery, Faculty of Medicine, McGill University, Montreal, QC, Canada; 5 MR Clinical Science, Philips Healthcare, Markham, ON, Canada; 6 Department of Medical Imaging, Division of Pediatric Radiology, Montreal Children’s Hospital, Montreal, QC, Canada; 7 Department of Pediatrics, Division of Cardiology, Montreal Children’s Hospital, Montreal, QC, Canada; 8 School of Physical & Occupational Therapy, McGill University, Montreal, QC, Canada; 9 Department of Pediatrics, Division of Neonatology, Montreal Children’s Hospital, Montreal, QC, Canada; University of Naples Federico II, ITALY

## Abstract

Congenital heart disease (CHD) has been associated with structural brain growth and long-term developmental impairments, including deficits in learning, memory, and executive functions. Altered functional connectivity has been shown to be altered in neonates born with CHD; however, it is unclear if these early life alterations are also present during adulthood. Therefore, this study aimed to compare resting state functional connectivity networks associated with executive function deficits between youth (16 to 24 years old) with complex CHD (mean age = 20.13; SD = 2.35) who underwent open-heart surgery during infancy and age- and sex-matched controls (mean age = 20.41; SD = 2.05). Using the Behavior Rating Inventory of Executive Function–Adult Version questionnaire, we found that participants with CHD presented with poorer performance on the inhibit, initiate, emotional control, working memory, self-monitor, and organization of materials clinical scales than healthy controls. We then compared the resting state networks theoretically corresponding to these impaired functions, namely the default mode, dorsal attention, fronto-parietal, fronto-orbital, and amygdalar networks, between the two groups. Participants with CHD presented with decreased functional connectivity between the fronto-orbital cortex and the hippocampal regions and between the amygdala and the frontal pole. Increased functional connectivity was observed within the default mode network, the dorsal attention network, and the fronto-parietal network. Overall, our results suggest that youth with CHD present with disrupted resting state functional connectivity in widespread networks and regions associated with altered executive functioning.

## Introduction

Congenital heart disease (CHD) refers to the presence of structural malformation(s) of the heart walls, valves, main blood vessels, and their relationships, resulting in impaired blood flow. With an incidence of 0.85% live births per year in Canada, CHD is the most common neonatal defect [1]. The standard of care practice for most complex CHD lesions is to perform open-heart surgery utilizing cardiopulmonary bypass during infancy, resulting in a significant improvement in life expectancy [2]. Although these individuals are now expected to live well into adulthood, a large variety of neurodevelopmental impairments are reported during childhood and adolescence. Among these, difficulties with language, social cognition, and higher order cognitive abilities have been widely reported [3–6]. Moreover, the emerging literature in older children, adolescents, and adults with CHD converge in reporting specific difficulties with executive functions associated with poorer psychosocial health status and quality of life [3–5, 7, 8].

It is now well recognized that CHD impacts brain development during the antenatal and neonatal periods [9]. Magnetic Resonance Imaging (MRI) has contributed to our understanding of cerebral pathophysiological mechanisms in CHD. Indeed, a growing body of quantitative structural MRI studies have reported the presence of regional alterations in brain development in adolescents and young adults with complex CHD [10]. Differences reported include smaller volumes or morphometric variations in the cortical and subcortical grey matter [3, 11–13], as well as microstructural alterations predominantly in the association tracts and frontal regions [14, 15]. Moreover, previous findings have reported associations between regional structural alterations and cognitive functions; however, these relationships were generally small in magnitude [3, 13, 16].

When examining brain functional connectivity in CHD, results are scarce. Resting state functional MRI (rs-fMRI) is a neuroimaging technique that evaluates regional brain interactions occurring while the participant is at rest (i.e., not performing any task). This technique allows the investigation of resting state networks, which provide information about inherent brain function during normal development or following injury. Resting state fMRI uses the blood-oxygen-level-dependent (BOLD) signal to measure spontaneous low frequency fluctuations (<0.1 Hz) in absence of a stimulus. This technique allows for the exploration of synchronous activations between different brain regions to describe networks, known as Resting State Networks, that can be reproducible across subjects [17]. Since the discovery of resting state networks, rs-fMRI studies have provided new insights into the understanding of typical and atypical brain functioning at rest when studying various pediatric and adult brain pathologies, including autism spectrum disorder, schizophrenia, and Alzheimer’s disease [18–20]. To the best of our knowledge, only one study to date has examined functional connectivity using rs-fMRI in individuals with CHD. The authors reported that neonates with complex CHD, prior to open-heart surgery, presented with preserved global functional network organization, but altered regional functional connectivity, when compared to healthy controls [21]. More precisely, altered functional connectivity was found in subcortical regions, including the putamen, caudate nucleus, globus pallidus, and thalamus, and in various cortical regions, especially in frontal, parietal, and temporal areas. Although this first study provides valuable insight into functional network topology and regional functional connectivity, it remains unknown if these functional connectivity deficits remain present beyond the post-surgical period. Moreover, whether these connectivity alterations are associated with later-developing cognitive functions remains to be determined. The current study aimed to fill these gaps. To do so, we sought to compare functional connectivity between youth with complex CHD who had undergone open-heart surgery during infancy and healthy peers, targeting networks associated with at-risk executive functions. We hypothesized that youth with complex CHD would exhibit altered functional connectivity in networks associated with executive functioning. As a secondary objective, we also explored the direct relationships between functional network connectivity and altered executive functions. A better understanding of the neural correlates of cognitive difficulties in youth with complex CHD will provide insight into the development and implications of the disease.

## Materials and methods

### Participants

French- and English-speaking youth aged 16 to 24 years old born with complex CHD who underwent open-heart surgery (OHS) using cardiopulmonary bypass during the first year after birth were enrolled in this study. Participants born preterm (<37 weeks of gestation), with documented congenital infection, a known chromosomal or genetic abnormality, or multiorgan dysmorphic features were excluded. Participants with CHD were recruited from the pediatric and the adult cardiology units of the McGill University Health Center (MUHC) as previously reported [13].

A control group, matched for age and sex, was recruited from local colleges, universities, and the community through advertisements and word of mouth. Controls were considered healthy if they had no history of brain tumor or malformation, traumatic brain injury, developmental or neurologic conditions and had not received rehabilitation or special education services during childhood or adolescence. Written informed consent was obtained from the participant, or legal guardians when younger than 18 years old. The study was approved by the MUHC Pediatric Research Ethics Board.

### Individual and clinical variables

All the participants underwent a single study visit at the Montreal Children’s Hospital to complete a brain MRI. Height and weight were measured before the MRI to compute body mass index (BMI). Socioeconomic status (SES) was measured using the Hollingshead Four-Factor Index questionnaire [22] and relevant clinical information, such as cardiac diagnosis, age at first surgery, and number of open-heart surgeries, was extracted from the medical records of the CHD participants.

### Executive function and self-regulation

On the day of the MRI, participants completed the Behavior Rating Inventory of Executive Function–Adult Scale (BRIEF-A) a norm-referenced, self-reported questionnaire that evaluates executive function and self-regulation [23]. This test is composed of nine clinical scales: inhibit, shift, emotional control, self-monitor, initiate, working memory, plan/organize, task monitor, and organization of materials, which together provide a total score for metacognition and behavioral regulation. On the BRIEF-A, higher scores represent poorer executive functioning.

### MRI data acquisition

Participants underwent a single brain MRI on a 3.0 T MRI (Achieva X-series, Philips Healthcare) using a 32-channel head coil. The acquisition protocol included three-dimensional 1 mm isotropic T1-weighted images (TE = 3.7 ms, TR = 8.1 ms, TI = 1010 ms, pixel bandwidth = 191.4 Hz/pixel, FOV = 240x240 mm, slice thickness = 1 mm, flip angle = 8°) and a gradient-echo echo planar imaging (EPI) sequence (TE = 30 ms, TR = 2600ms, pixel bandwidth = 2197.48 Hz/pixel, FOV = 240x240 mm, acquisition matrix = 80x80, slice thickness = 3 mm, flip angle = 70°, 47 slices/volume, interleaved, no gap). During the resting-state sequence, participants were awake and instructed to keep their eyes closed. The anatomical images were reviewed for overt brain anomalies by an experienced neuroradiologist, who was blinded to the clinical history of the participants.

### MRI data processing

Before pre-processing, T1-weighted and EPI images were visually inspected for possible artifacts (e.g., spikes, signal loss, aliasing). Spatial pre-processing of images was first performed and included the following steps: functional cross-realignment for head motion correction, slice timing correction, outlier scrubbing [24], tissue segmentation, normalization to Montreal Neurological Institute (MNI) 152 space, and outlier detection using the Artifact Detection Tool (ART) (www.nitrc.org/projects/artifact_detect). Total grey matter masks before normalization were used to examine grey matter volume differences between the two groups. Afterwards, data were spatially smoothed with an 8 mm full-width half-maximum gaussian kernel [25].

We used a gaussian kernel of 2–3 times the voxel size, as this has been shown to be optimal to correct for truncation artifacts [26], to increase signal-to-noise ratio, and to reduce the influence of residual variability and gyral anatomy across subjects. After spatial pre-processing, realigned images in MNI space were visually inspected by overlapping the MNI structures over structural and functional data to confirm optimal realignment.

The BOLD signal of interest may be altered by macro vessel signal, mainly those located in the pial surface, as well as by some non-physiological signals, such as head motion. To correct for these nuisance variables, temporal processing was performed using a component-based noise correction method [27–29]. From the T1-weighted images, white matter and cerebrospinal fluid were segmented using SPM12 (www.fil.ion.ucl.ac.uk/spm) and used in a subsequent step to remove the temporal confounding factors. Nuisance variables were based on cerebrospinal fluid signal, white matter signal, motion, and realignment parameters [28]. Potential outliers were identified from subject motion and observed global BOLD signal using ART. Volumes with framewise displacement higher than 0.5 mm and/or that presented global signal changes above 3 standard deviations were identified as outliers. The anatomical CompCor method was used for nuisance correction [27, 30], as it has been shown to be as efficient as global signal regression-based methods, but without inducing undue anti-correlations [27, 31]. Functional data were linearly detrended and band-pass filtered [0.008–0.09Hz] to adjust for low frequency fluctuations related to very slow head displacements, scanner-related drifts, and high frequency noise effects [32–35].

A quality control plot was created to detect outliers after denoising, consisting of a voxel-to-voxel correlation histogram. Before denoising, functional connectivity distribution values within the whole brain showed highly positively skewed distributions and appeared to be very different across subjects due to the influence of large-scale physiological signals and head motion effects. After having corrected for the aforementioned confounders, functional connectivity distributions appeared to be well centered and very similar across subjects, suggesting that the noise effect had been appropriately removed [S1 Fig]. To achieve the desired denoising data quality, we employed for every subject: white matter (5 components), cerebrospinal fluid (5 components), scrubbing (one per identified outlier volume), motion (6 components + 1^st^ order derivatives). After this step, as the degrees of freedom of every participant were still high, we decided to include the quadratic effects to the realignment component to improve motion related denoising. Finally, we used the number of degrees of freedom remaining after the denoising process as an exclusion factor, excluding subjects with less than 15 degrees of freedom. All the participants presented with centered and normalized data and enough degrees of freedom to be analyzed.

To process and analyze the resting state fMRI data, we used the CONN Toolbox 18.b (http://nitrc.org/projects/conn), based on SPM12 and running in MATLAB R2018a (MathWorks, Inc, Natick, MA, USA) on an Ubuntu 18.04 machine.

### Seed selection

Seed-based functional connectivity (SBC) analysis was performed to identify differences in brain functional connectivity between the CHD and control groups. Considering the increasingly recognized functional challenges reported in adolescents and young adults with CHD, we chose to use a hypothesis-driven approach over a data-driven approach to compare functional connectivity in networks related to executive function as identified by the BRIEF-A. Seeds for each region and network were placed using the probabilistic Harvard-Oxford atlas (http://neuro.debian.net/pkgs/fsl-harvard-oxford-atlases.html). The selected seeds corresponded to brain regions or networks known to be involved in executive functions that were found to be significantly different in our group comparisons of the BRIEF-A clinical scales. In line with these findings, we analyzed the default mode network (internal modes of cognition) [36], the dorsal attention network (attentional capabilities) [37], the fronto-parietal [38] and fronto-orbital networks (high-level cognition function) [39, 40], and the amygdalar network (emotional control) [41, 42]. To assess the different networks, seeds were placed as specified in the Conn toolbox [32]. The medial prefrontal cortex (mPFC) and the posterior cingulate cortex (PCC) were chosen to assess the default mode network (DMN), the intra-parietal sulcus (IPS) and the frontal eye fields (FEF) to assess the dorsal attention network (DAN), and the lateral prefrontal cortex (LPFC) and the parietal cortex (PPC) to assess the fronto-parietal network (FPN). The fronto-orbital network was added to the analyses given its link with executive functions and the amygdalar network was added for playing an important role in emotional control, with seeds placed in the fronto-orbital cortex and in the amygdala, respectively.

### Statistical analysis

#### Participants’ characteristics and BRIEF-A scores

Participants’ characteristics were compared between the CHD and control groups using independent sample t-tests or chi-square tests, as appropriate. Variables that showed significant group differences were considered potential confounders and included in subsequent analyses. Analyses of covariance (ANCOVA) were performed for each clinical scale of the BRIEF-A, using potential confounders as covariates when relevant. The only significantly different confounder between the CHD and control groups was socioeconomic status, which was included as a covariate in subsequent analyses. In all analyses, the alpha level was set at 0.05.

#### Resting state functional connectivity

The average time series of each single seed was computed across each seed region in each participant, and then correlated with the time series of every other voxel in the brain. Correlation maps were calculated using the standard Pearson product-moment formula as described in Biswal et al. [43]. Correlation coefficients were normalized by Fisher’s z-transformation.

Group differences in functional connectivity between CHD and controls were assessed using ANCOVA, using socioeconomic status as a covariate. Statistical significance between groups was established as p < 0.001 (uncorrected) at the voxel level and as p < 0.05 (corrected for family wise error [FWE]) at the cluster level [44, 45].

Additionally, to explore the direct associations between network functional connectivity and executive function deficits, we performed two-tailed Pearson correlations between functional connectivity and BRIEF-A scores, focusing on the BRIEF-A scales previously identified to be significantly different between the two groups. These correlation analyses were performed separately in the control and CHD groups. The level of statistical significance as set at p < 0.05. We did not correct for multiple comparisons considering the exploratory nature of these analyses.

## Results

### Participants’ characteristics

In total, we collected 43 rs-fMRI acquisitions in the CHD group and 47 in the control group. Of these, five participants from the CHD group and two from the control group were excluded from the analysis for not having a complete rs-fMRI acquisition. Another CHD participant was excluded from the analysis for not passing the aforementioned quality assessment. Our final sample for analysis consisted of 37 participants with CHD (14/37 male) and 45 controls (19/45 male). Of the 37 participants with CHD, 32 (86.49%) presented with a two-ventricular cardiac physiology: dextro-transposition of the great arteries (n = 13), Tetralogy of Fallot (n = 10), total anomalous pulmonary venous connection (n = 2), ventricular and atrial septal defects (n = 5), and truncus arteriosus type I (n = 2). Only 5/37 (13.51%) presented with a univentricular physiology: double outlet right ventricle (n = 1), pulmonary atresia (n = 3), and hypoplastic left heart syndrome (n = 1). CHD participants had between one and four open-heart surgeries (median 1) at the time of the study visit. Mean age at first surgery was 68.65 days old, with a range from zero to 293 days after birth. Socioeconomic status was found to be significantly higher in the control group when compared to the CHD group and was consequently included as a covariate in subsequent analyses. No other significant group differences were found for age at MRI, sex, and BMI [Table 1].

**Table 1:** Participants’ characteristics.

**Variables; mean [SEM], N (%)**	**CHD (n = 37)**	**CTL (n = 45)**	**p-value**
Age at MRI, years	20.13 [0.38]	20.41 [0.30]	0.34
Age first surgery, days	68.65 [15.51]	-	-
Sex			0.80
Male	14 (37.8%)	19 (42.2%)	
Female	23 (62.2%)	26 (57.8%)	
Body mass index	23.31 [0.67]	23.92 [0.56]	0.23
Grey matter volume, dm^3^	0.25 [0.015]	0.26 [0.014]	0.36
Socioeconomic status	39.95 [2.07]	50.73 [1.54]	<0.001
Type of CHD			
Single ventricle	5/37 (13.5%)	-	-
Tetralogy of Fallot	10/37 (27.0%)	-	-
Transposition of great arteries	13/37 (35.1%)	-	-
Other two-ventricle physiology			
Ventricular/atrial septal defects	5/37(13.5%)	-	-
Truncus arteriosus type I	2/37(5.4%)	-	-
Total anomalous pulmonary	2/37(5.4%)	-	-
venous connection			
Total surgery time (min)	134.5 [8.57]	-	-
Aortic cross clamp time (min)	76.59 [6.09]	-	-
Deep hypothermia time (min)	19.28 [4.06]	-	-
Catheterizations	22/32 (68.75%)	-	-
Balloon atrial septostomy before surgery	8/15 (53.33%)	-	-

CHD: congenital heart disease, CTL: control, SEM: standard error of the mean.

Brain anomalies on conventional MRI likely from an acquired origin were detected in 7/37 (18.92%) CHD participants and 4/45 (8.89%) of the controls, which was not statistically different (p = 0.210) These anomalies included: One CHD participant with cystic dilation of the perivascular spaces; two CHD participants with periventricular white matter injury; two CHD participants and three controls with susceptibility artifact, likely representing blood deposition or calcification; and two CHD participants and one control with asymmetrical ventricles. Brain anomalies likely from a developmental origin were found in 6/37 (16.22%) CHD participants and 2/45 (4.44%) controls, (p = 0.123). These anomalies included: Three CHD participants and one control with grey matter heterotopia; two CHD participants and two controls with developmental venous anomalies; one CHD participant with cortical developmental anomaly; and one CHD participant with Chiari I malformation. The observed anomalies were all considered to be mild and from a remote origin, and none of the brain anomalies detected on conventional MRI overlapped with any of the connectivity networks analyzed.

### Executive functions

After controlling for SES, participants with CHD demonstrated significantly poorer performance than control participants on the inhibit (F(1,76) = 7.16; p = 0.009), emotional control (F(1,76) = 7.24; p = 0.009), self-monitor (F(1,76) = 7.09; p = 0.009), initiate (F(1,76) = 4.22; p = 0.04), working memory (F(1,76) = 5.25; p = 0.025), and organization of materials (F(1,76) = 18.51; p<0.001) clinical scales of the BRIEF-A. By contrast, there were no significant differences between groups for the shift (F(1,76) = 1.21; p = 0.27), plan/organize (F(1,76) = 3.40; p = 0.07), and task monitor (F(1,76) = 3.53; p = 0.06) clinical scales. Scores for BRIEF-A scales are summarized in Table 2. Participants were classified as having a clinically significant deficit on a given clinical scale when having a score ≥ 65. On average, a greater percentage of participants with CHD had clinically significant executive function deficits as compared to controls (6–40% in CHD vs. 0–13.6% in controls) across the different clinical scales. Differences in the prevalence of clinically significant deficits were statistically significant for the inhibit (χ^2^ = 14.23; p < 0.001), working memory (χ^2^ = 7.1; p = 0.008), and organization of materials (χ^2^ = 11.09; p < 0.001) clinical scales (S1 Table).

**Table 2:** BRIEF-A scales results.

Mean (SEM)	CHD (n = 35)	CTL (n = 44)	p-value
Inhibit**	55.6 (2.21)	50.4 (2.04)	0.009
Shift	53.6 (2.15)	51.5 (2.41)	0.27
Emotional control**	56.9 (1.93)	49.5 (2.57)	0.009
Self-monitor**	54.1 (2.28)	46.5 (2.19)	0.009
Initiate*	54.9(1.80)	51.2 (2.42)	0.04
Working memory*	59.1 (2.11)	53.0 (2.34)	0.02
Plan/ Organize	53.5 (1.62)	50.5 (1.93)	0.07
Task monitor	57.0 (2.02)	52.8(2.86)	0.06
Organization of materials***	55.2 (2.19)	46.6 (2.76)	<0.001

* p < 0.05

** p < 0.01

***p < 0.001. NB: Higher scores of BRIEF-A indicate poorer performance.

### Resting state functional connectivity

We performed comparisons of seed-based functional connectivity between the two groups for the default mode, dorsal attention, fronto-parietal, fronto-orbital, and amygdalar networks. For each analysis, the threshold of statistical significance as set to p < 0.001 (uncorrected) at the voxel level and p < 0.05 (corrected for family wise error [FWE]) at the cluster level. Voxels that did not survive the threshold were not displayed.

#### Decreased functional connectivity

**Fig 1:**
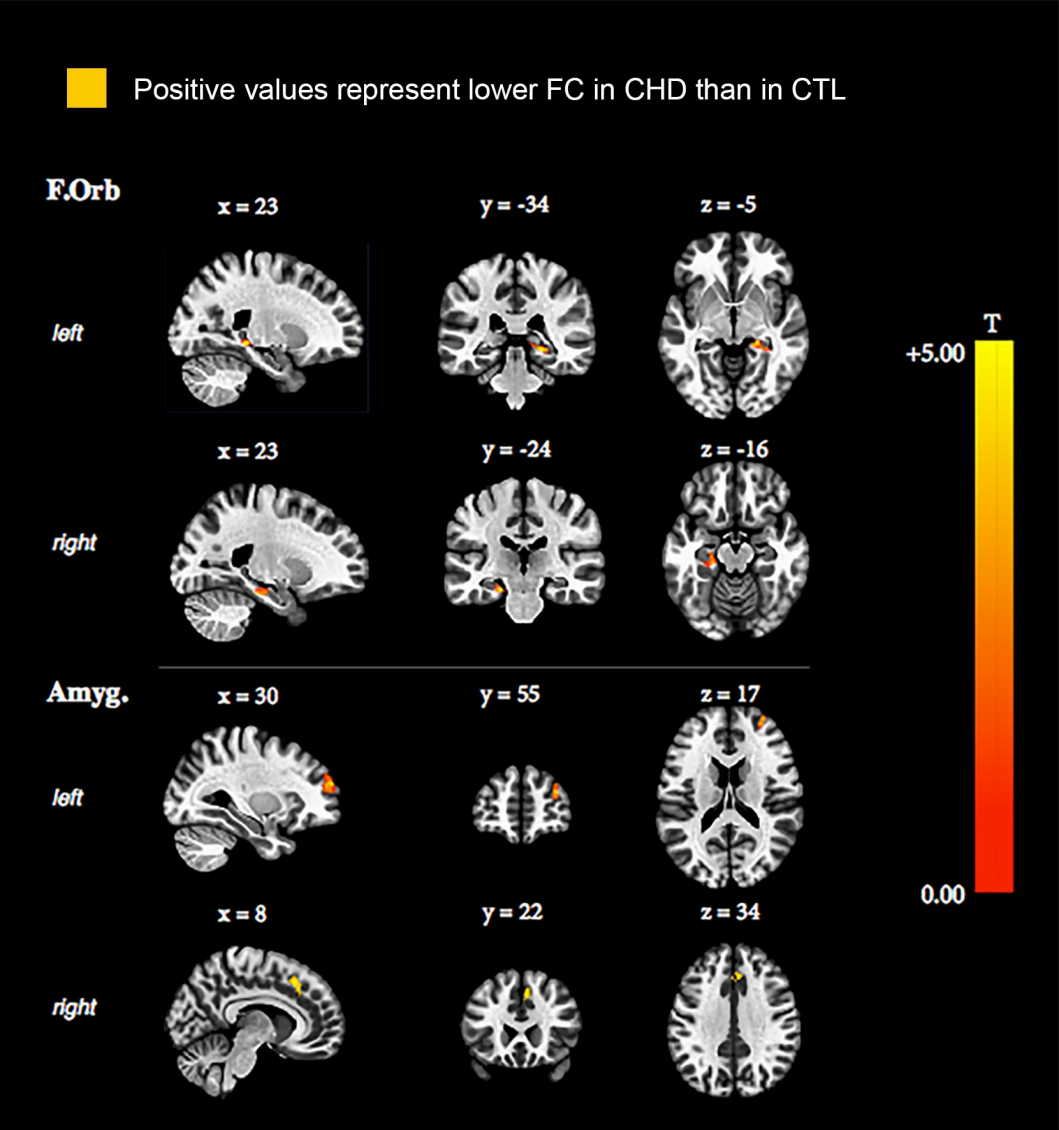
Functional connectivity differences between CHD and control groups where CHD presented lower functional connectivity. CHD: congenital heart disease, CTL: controls, F.Orb: fronto-orbital network, Amyg: amygdalar network.

*Fronto-orbital network*. Our analyses revealed lower inter-network functional connectivity in the CHD group when compared to controls between the right fronto-orbital cortex and the left hippocampus and between the left fronto-orbital cortex and bilateral hippocampi [Fig 1, Table 3].

**Table 3:** Functional connectivity differences between CHD and control groups where CHD participants presented with lower functional connectivity than controls.

	Affected region	Cluster size	Peak p-uncorrected	Cluster p<FWE
**CHD lower FC than CTL**				
F.Orb (right cortex)	Left hippocampus	113	<0.001	0.03
F.Orb (left cortex)	Right and left hippocampus	185	<0.001	<0.01
Amyg (left amygdala)	Frontal pole right	270	<0.001	<0.001
Amyg (left amygdala)	Cingulate and paracingulate gyrus right	134	<0.001	0.01

FC: functional connectivity, CHD: congenital heart disease, CTL: controls, F.Orb: fronto-orbital network, Amyg: amygdalar network.

*Amygdalar network*. We found significantly lower inter-network functional connectivity in the CHD group compared to controls between the left amygdala and the right frontal pole and between the left amygdala and the right cingulate and paracingulate gyrus [Fig 1, Table 3].

### Increased functional connectivity

**Fig 2:**
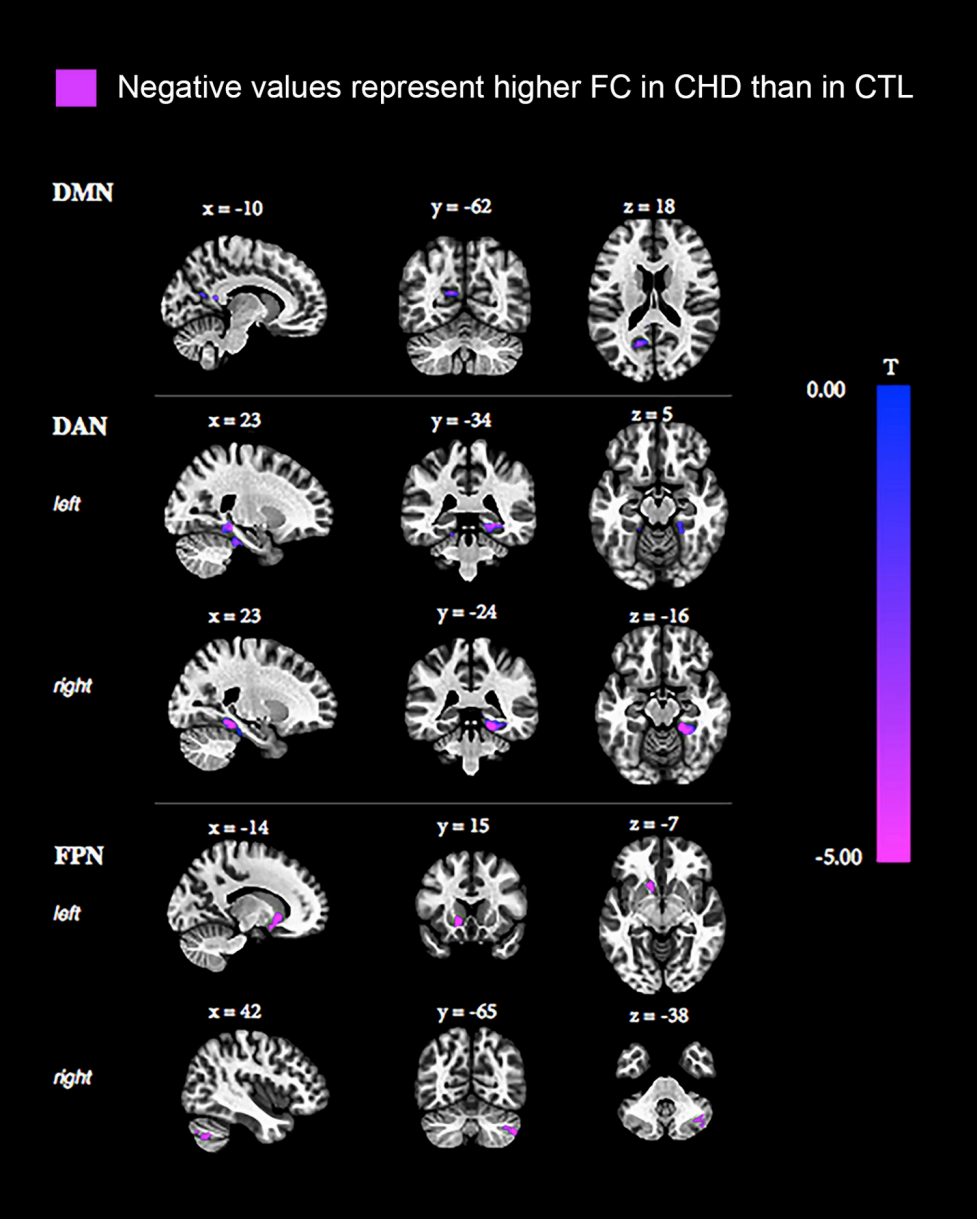
Functional connectivity differences between CHD and control groups where CHD participants presented with higher functional connectivity than controls. CHD: congenital heart disease, CTL: controls, DMN: default mode network, DAN: dorsal attention network, FPN: fronto-parietal network.

*Default mode network*. When compared to controls, participants with CHD presented with higher intra-network functional connectivity between the medial prefrontal cortex and the posterior cingulate cortex [Fig 2, Table 4].

**Table 4:** Functional connectivity differences between CHD and control groups where CHD participants presented with higher functional connectivity than controls.

	Affected region	Cluster size	Peak p-uncorrected	Cluster p<FWE
**CHD higher FC than CTL**				
DMN (medial prefrontal cortex)	Posterior cingulate cortex	133	<0.001	0.018
DAN (intraparietal sulcus left)	Right hippocampal and parahippocampal regions	405	<0.001	<0.001
DAN (intraparietal sulcus left)	Left hippocampal and parahippocampal regions	263	<0.001	<0.001
DAN (intraparietal sulcus right)	Right hippocampal and parahippocampal regions	348	<0.001	<0.001
FP (parietal cortex left)	Left caudate, left accumbens and left putamen	161	<0.001	0.01
FP (parietal cortex left)	Cerebellum Crus 1–2 right	151	<0.001	0.01

FC: functional connectivity, CHD: congenital heart disease, CTL: controls, DMN: default mode network; DAN: dorsal attention network; FPN: fronto-parietal network.

*Dorsal attention network*. Similar to the DMN, our analyses showed significantly higher inter-network functional connectivity in participants with CHD when compared to controls between the left intraparietal sulcus (ips_l) and the bilateral hippocampal and parahippocampal regions, but also between the right intraparietal sulcus (ips_r) and the right hippocampal and parahippocampal regions [Fig 2, Table 4]. No significant differences were found for the frontal eye fields.

*Fronto-parietal network*. We observed significantly higher inter-network functional connectivity in the CHD group when compared to controls between the left parietal cortex (ppc_l) and the left caudate, left accumbens, and left putamen, and between the ppc_l and the right crus of the cerebellum [Fig 2, Table 4]. No significant differences were found for the lateral prefrontal cortex.

### Correlations between functional connectivity and BRIEF-A scores

We observed two different sets of significant correlations between functional connectivity and BRIEF-A scores in the CHD participants and in the control participants. In the CHD group, significant correlations were found between right frontal orbital–left hippocampus functional connectivity and the inhibit scale (r = -0.36, p = 0.03), as well as between medial prefrontal cortex–posterior cingulate functional connectivity and the organization of materials scale (r = 0.35, p = 0.038). In the control group, significant correlations were observed between left intraparietal sulcus–right hippocampus functional connectivity and the emotional control scale (r = 0.36, p = 0.014), as well as between left amygdala–cingulate/paracingulate gyrus functional connectivity and the organization of materials scale (r = -0.35, p = 0.019*)*.

## Discussion

The present study investigated cerebral functional connectivity in brain networks associated with executive functioning in youth born with complex CHD who had undergone open-heart surgery using cardiopulmonary bypass during infancy. Our evaluation of function-specific resting state networks revealed statistically significant differences in functional connectivity in youth born with complex CHD, when compared to healthy peers, with significant alterations of functional connectivity within the fronto-orbital cortex, amygdala, default mode, dorsal attention, and fronto-parietal networks.

To the best of our knowledge, this is the first resting state functional connectivity study on post-operative CHD patients. The only other previous study of resting state functional connectivity in this population was performed in neonates with complex CHD prior to open-heart surgery [21]. This prior study reported that pre-operative CHD neonates exhibited reduced rich club network organization in functional brain network connectivity when compared to healthy term-born neonates, as well as reduced sub-network connectivity, predominantly implicating the subcortical areas, such as the caudate, putamen, and thalamus, and their connections to the contralateral frontal, parietal, and temporal cortices. They also reported reduced functional connectivity within the hippocampus and other brain structures, in line with our current findings. Indeed, we found decreased functional connectivity in youth with CHD between the fronto-orbital network and the hippocampus. Taken together, these observations may suggest that alterations in hippocampal functional connectivity that are present prior to open-heart surgery likely persist during development and after cerebral hemodynamics have been restored following cardiac surgery. Our results also converge with previous findings from anatomical studies that have demonstrated smaller hippocampal volumes and morphometric differences in adolescents and young adults with CHD, associated with poorer memory and executive functioning [3, 13]. Future fMRI studies using the hippocampal structural input as a seed in network analysis could clarify the relationship between the structural alterations and functional connectivity alterations reported in youth with CHD.

Participants with complex CHD in our study also presented with decreased functional connectivity between the amygdala and frontal and cingulate regions. The amygdala’s interaction with the frontal cortex plays a crucial role in the regulation of emotion [46], known to be vulnerable in CHD survivors and found to be significantly different from controls on the associated BRIEF-A clinical scale. Elevated rates of anxiety disorders, attention deficit hyperactivity disorder, and depression have been reported in adolescents and adults with complex CHD [47, 48], which could theoretically be related to amygdala dysfunction. In adolescents with major depressive disorders, studies have previously shown decreased amygdala functional connectivity [49, 50]. A recent study reported that adults with complex CHD are more likely than controls to present with some personality traits, in particular neuroticism (i.e., experiencing emotional negativity and instability) [51]. However, whether these psychiatric symptoms are present in youth with CHD and are related to altered functional amygdalar connectivity will need to be further investigated, considering that we did not specifically evaluate mental health.

An unexpected finding was the observation of increased functional connectivity of the DMN in participants with CHD when compared to controls. The DMN has been extensively studied in the healthy populations and in various psychiatric, neurological, and neurodevelopmental conditions [36, 44, 52–54]. While decreased functional connectivity within the DMN has been widely reported in pathologies such as autism spectrum disorder, schizophrenia, and Alzheimer’s disease [55], an increased functional connectivity between the mPFC and the PCC has been demonstrated in patients with mild cognitive impairment [53]. This is particularly of interest considering that there is emerging literature suggesting that seniors living with CHD are at greater risk of early onset dementia [56, 57]. Moreover, the DMN is engaged in self-referential (internal) thinking and is disengaged during attentional-demanding (external) processes [54]. Thus, greater activity within the DMN when individuals are at rest is thought to be reflective of difficulties in switching from internal to external thoughts [58]. In participants with CHD, this may reflect their cognitive and attentional dysfunction in daily life [59–61], as they may experience difficulties switching from internal to external stimulation. However, considering that we have not evaluated attention and hyperactivity, this hypothesis remains speculative. Similarly, altered amygdalar connectivity, combined with DMN dysfunction, may underlie in part some of the difficulties in regulating emotions or internal states that are frequently observed in individuals with CHD [52].

Participants with CHD also demonstrated increased functional connectivity in the dorsal attention network and the fronto-parietal network when compared to controls. The dorsal attention network is known to be engaged during externally directed attentional tasks and its activity is increased when individuals must focus their attention on external stimuli. Its level of activity is thought to reflect and predict attentional skills [37]. Although we did not evaluate attention specifically, participants with CHD presented with lower scores on the organization of materials scale, likely reflecting difficulty when handling more than one stimulus at the same time, which is driven in part by attentional skills. Regarding the fronto-parietal network differences, increased resting state functional connectivity was specifically observed in the CHD group between the parietal cortex and the cerebellar Crus 1 and Crus 2 regions. Theses cerebellar regions are known to be involved in executive functions, coherent with our findings [62].

When performing correlations between functional connectivity and BRIEF-A scores, we observed different modest correlations in the two groups. Interestingly, we observed a negative correlation between amygdala–cingulate/paracingulate gyrus connectivity and the organization of materials scale in the control group, while a positive correlation between the default mode intra-network functional connectivity and this scale was detected in the CHD group. Higher levels of inhibition have been shown to correlate with decreased amygdala–cingulate functional connectivity [63]. However, we could not find previous reports of a relationship of organizational cognitive tasks with amygdala–cingulate or default mode network functional connectivity. The lack of strong associations in these exploratory analyses may have been mitigated by the use of a self-reported questionnaire to measure executive functioning, which may have induced some bias. Although the BRIEF-A has been demonstrated to be valid and reliable for measuring executive function in various clinical populations [23], standardized batteries may be more objective in examining a wider range of higher-order cognitive performance.

Abnormalities in functional connectivity may reflect alterations of the structural organization of white matter tracts. Indeed, several diffusion tensor imaging studies have detected lower fractional anisotropy in adolescents with CHD [14, 15, 64–66]. These findings may reflect potential alterations to numerous facets of white matter microstructure, including alterations to myelination, axon density, axon diameter, axon orientation, or cell membrane permeability [67]. Additionally, we recently applied neurite orientation dispersion and density imaging, an advanced diffusion MRI modelling technique, in this cohort of CHD survivors, detecting widespread reductions in the neurite density index, reflecting a lower density of axon packing [14]. Altered regions included frontal and limbic white matter tracts, in line with the altered functional connectivity we describe in these regions. Nevertheless, future multi-modal MRI studies combining structural and functional connectivity analyses are needed to disentangle these complex relationships.

### Limitations

Our results should be considered within the context of their limitations. It is important to highlight that our sample of CHD participants included a mixed cohort of different CHD physiologies, and therefore cannot be generalized to a specific subtype. Nevertheless, the fact that we included participants with a variety of complex CHD physiologies operated during infancy is representative of the clinical diversity of this condition. Also, we recognize that the use of atlas-based seed selection may have introduced a potential bias [68]. Future studies using data driven approach such as independent component analyses may provide complementary information to the current findings. Additionally, it is important to consider that differences in brain volume and morphometry are common in individuals with CHD as compared to healthy peers [10, 13], which theoretically could have influenced our resting state fMRI results. However, considering that total grey matter volume did not differ between the groups in our sample and that we used a validated pre-processing pipeline that included non-linear registration [69] to overcome this potential effect, we believe this risk to be minimal in the current sample. Lastly, the differences found in socioeconomic status between the two groups may be a limitation of the study; however, we carefully corrected for this potential confounder in our analyses.

## Conclusion

The current study provides the first evidence supporting the presence of altered functional connectivity in youth born with complex CHD. Specifically, we found atypical functional connectivity in youth with CHD in the fronto-orbital cortex, amygdala, default mode, dorsal attention, and fronto-parietal networks. In this new era of open-science, future studies using longitudinal imaging in large multi-center cohorts will strengthen our understanding of long-term altered connectivity and how to measure the risk for these alterations at an individual level, in order to better identify at-risk children and adolescents that could benefit from targeted interventions.

## Supporting information

S1 Fig Functional connectivity distribution before and after denoising. (TIF)

S1 Table Proportion of participants who performed below clinical cut-off on the BRIEF-A. Overall, a greater percentage of youths with CHD performed below scale’s clinical cutoff (i.e., >65) than control (5.7–40.0% in CHD vs. 0–13.6% in controls) and reaching statistical significance level for three subscales: inhibit (X^2^ = 14.23; p< 0.001), working memory (X^2^ = 7.1; p = 0.008) and organization of material (X^2^ = 11.09; p< 0.001).
(PDF)
