# Supplementary figures and images for: Altered resting state functional connectivity in youth with congenital heart disease operated during infancy
Source: PLoS One. 2022 Apr 15;17(4):e0264781. doi: 10.1371/journal.pone.0264781 (PMC9012393; doi:10.1371/journal.pone.0264781)

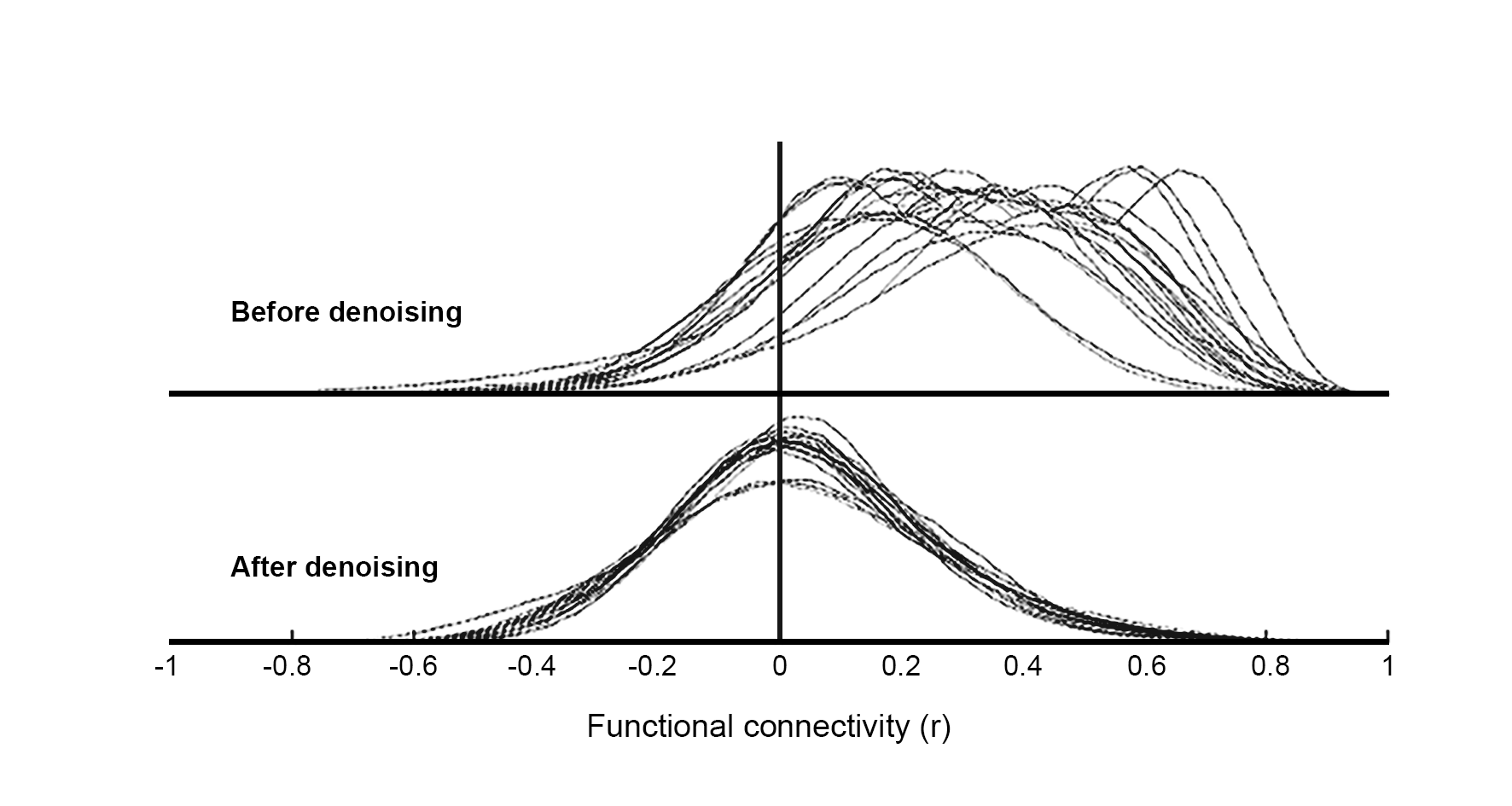

Supplement: S1 Fig — (TIF) [file pone.0264781.s001.tif]
