# Supplementary material for: Altered resting state functional connectivity in youth with congenital heart disease operated during infancy
Source: PLoS One. 2022 Apr 15;17(4):e0264781. doi: 10.1371/journal.pone.0264781 (PMC9012393; doi:10.1371/journal.pone.0264781)
Supplement: S1 Table — Overall, a greater percentage of youths with CHD performed below scale’s clinical cutoff (i.e., >65) than control (5.7–40.0% in CHD vs. 0–13.6% in controls) and reaching statistical significance level for three subscales: inhibit (X2 = 14.23; p< 0.001), working memory (X2 = 7.1; p = 0.008) and organization of material (X2 = 11.09; p< 0.001). (PDF) [file pone.0264781.s002.pdf]

Supplementary Table 1

N (%)	CHD (n=35)	CTL (n=44)	p-value
Inhibit***	10 (28.6)	0.0 (0.0)	< 0.001
Shift	6 (17.1)	4 (9.1)	0.29
Emotional control	6 (17.1)	4 (9.1)	0.29
Self-monitor	7 (20.0)	3 (6.8)	0.08
Initiate	6 (17.1)	5 (11.4)	0.47
Working memory**	14 (40.0)	6 (13.6)	0.008
Plan/ Organize	2 (5.7)	2 (4.5)	0.81
Task monitor	9 (25.7)	6 (13.6)	0.18
Organization of materials***	10 (28.6)	1 (2.3)	< 0.001
